# “Laboratorium Repository”: a training tool for the Healthcare Workforce: Guglielmo Arzilli

**DOI:** 10.1093/eurpub/ckac130.144

**Published:** 2022-10-25

**Authors:** GP Privitera, G Arzilli, C Di Benedetto, F Di Serafino, E Morassi, N Zotti, M Luzi, F Papini, V Casigliani, S Brusaferro

**Affiliations:** 1 Training Office, National Institute of Health, Rome, Italy; 2 Department of Translational Research and and of New Surgical and Medical Technologies, University of Pisa, Pisa, Italy; 3 Information Technology, National Institute of Health, Rome, Italy; 4 National Institute of Health, Rome, Italy

## Abstract

**Background:**

The COVID-19 pandemic highlighted the need to redefine the healthcare workforce (HCW) competencies to face future emergencies linked to emerging infectious diseases, environmental, climate and social crises. As recently stated by WHO, there is a need to identify standards for education and competencies training for HCW in emergency and preparedness (E&P). The Italian National Institute of Health, in agreement with the deliberation of the G20 Health Ministers under the Italian Presidency, is developing an educational program named “Laboratorium” which includes a free access digital repository aimed to share selected documents and tools at the International Public HCW (PHCW) to increase the competencies in E&P response.

**Objectives:**

A range of web domains selected according to their reliability was monitored using a keyword search tool for any relevant material published from February 14th up to April 28th, 2022. We included any publications, training materials, epidemiological data, initiatives, and communication items that addressed the topic of interest. Each item was submitted for approval by a scientific board and, if appropriate, classified by typology, language, topic, and country before publication.

**Results:**

To date, out of 6197 items, 418 fulfilled the inclusion criteria. For the type of content, we included guidelines/recommendations (75), epidemiological data (58), websites (34), online courses (15) and books (16). PHCW was the most representative target group (361), followed by other stakeholders (127), hospital practitioners (90), primary care (87). The most represented topic was infectious diseases/SARS-CoV-2 (277) followed by vaccines (88), emergency interventions (34), emerging diseases (17), policies (26), public health preparedness (32).

**Conclusions:**

Future training for PHCW should be designed with a modular approach with different levels of usability. The Laboratorium Repository provides a core of items for learning according to one's training needs

**Key messages:**

- The Laboratorium repository offers to PHCW a tool for updating their emergency and preparedness competencies.
- This repository has a user-friendly interface, accessible also through mobile devices.

